# Nutritional and Psychosocial Impact of Food Allergy in Pediatric Age

**DOI:** 10.3390/life14060695

**Published:** 2024-05-28

**Authors:** Luca Pecoraro, Carla Mastrorilli, Stefania Arasi, Simona Barni, Davide Caimmi, Fernanda Chiera, Giulio Dinardo, Serena Gracci, Michele Miraglia Del Giudice, Roberto Bernardini, Arianna Giannetti

**Affiliations:** 1 Pediatric Unit, Department of Surgical Sciences, Dentistry, Gynecology and Pediatrics, University of Verona, 37126 Verona, Italy; 2 Department of Pediatrics, University Hospital Consortium Corporation Polyclinic of Bari, Pediatric Hospital Giovanni XXIII, 70124 Bari, Italy; 3 Area of Translational Research in Pediatric Specialities, Allergy Unit, Bambino Gesù Children’s Hospital, IRCCS, 00165 Rome, Italy; 4 Allergic Unit, Department of Pediatric, Meyer Children’s Hospital, 50139 Florence, Italy; 5 Allergy Unit, CHU de Montpellier, Université de Montpellier, 34295 Montpellier, France; 6 IDESP, UMR A11, Université de Montpellier, 34093 Montpellier, France; 7 Department of Pediatrics, San Giovanni di Dio Hospital, 88900 Crotone, Italy; 8 Department of Woman, Child and General and Specialized Surgery, University of Campania ‘Luigi Vanvitelli’, 80133 Naples, Italy; 9 Pediatrics and Neonatology Unit, Maternal and Child Department, San Giuseppe Hospital, Azienda USL Toscana Centro, 50053 Empoli, Italy; 10 Pediatric Unit, IRCCS Azienda Ospedaliero-Universitaria di Bologna, 40138 Bologna, Italy; ariannagiannetti25@gmail.com

**Keywords:** IgE-mediated food allergy, nutritional complications related to food allergy, psychosocial complications related to food allergy, quality of life

## Abstract

Treatment of IgE-mediated food allergy involves avoiding the food causing the allergic reaction. In association, an action plan for allergic reactions is indicated, sometimes including self-injectable adrenaline. In addition to these dietary and medical implications, there are two equally important ones: nutritional and psychosocial. From a nutritional point of view, it is known that children suffering from food allergy have a growth delay in height and weight compared to their non-allergic peers. Specifically, this condition is directly related to the specific food excluded from the diet, the number of foods excluded and the duration of the elimination diet. From a psychosocial point of view, the child often cannot eat the foods other guests eat. Children with food allergy may perceive an aura of parental anxiety around their mealtime and may be afraid that what they eat could have harmful consequences for their health. Furthermore, children’s and their parents’ quality of life appears to be affected. The need to manage the allergy and the nutritional and psychosocial problems positions the pediatric nutritionist and the child neuropsychiatrist as support figures for the pediatric allergist in managing the child with food allergy.

## 1. Introduction

Food allergy (FA) is increasingly recognized as a significant public health issue, with documented prevalence rates on the rise in recent decades [1]. Studies have indicated that FA affects approximately 3–10% of children and can impact up to 10% of adults [2,3,4]. Despite advances in medical research, including the development of specific immunotherapy, the cornerstone of FA management is allergen avoidance and emergency action plans [5]. However, despite these measures, the prevalence of accidental ingestion of allergens is 73% [6], and while fatal reactions are extremely rare, they can still occur, underscoring the importance of continued vigilance and preparedness. Beyond the physical risks, FA imposes a substantial psychological burden on both affected individuals and their families [7]. Coping with the constant threat of exposure to allergens, managing dietary restrictions, and navigating social situations can significantly impact the emotional well-being of those with FA and their loved ones. Furthermore, dietary limitations secondary to FA can lead to malnutrition, decreased growth velocity (height/length and weight), and/or deficiencies in specific macro- and micronutrients [8]. In light of these multifaceted challenges, this review seeks to comprehensively assess both the nutritional and psychological implications of FA in children.

## 2. Nutritional Aspects

The patient with FA is at risk of insufficient energy intake due to inadequate substitution in the allergen elimination diet. The delayed growth observed in children with FA compared to their healthy peers is well-documented [9,10], a concern reflected in all the current guideline documents, which list poor growth as one of the symptoms associated with childhood FA [11,12,13,14]. Specifically, this growth delay is directly correlated with the number of foods eliminated from the diet and the duration of the elimination diet [8,15]. Additionally, any nutritional deficiency is related to the specific food excluded from the diet [16]. Pediatric clinicians should closely monitor the growth (height/length as well as weight and, up to 36 months of age, head circumference) of children with food allergies and refer these patients to a registered dietitian for evaluation [14,17]. Specifically, height/length may be affected, with 1 prospective international survey of over 430 children finding stunting (height-for-age Z-score of <−2) to be more common than poor weight gain in children with food allergies [18]. Considering that cow’s milk and eggs are the most commonly implicated foods in the etiology of pediatric food allergy [19], the nutritional adequacy of a diet devoid of these foods can be problematic, as they provide a significant portion of nutrients in a child’s diet and are present in various foods, thus complicating dietary choices for the child [16].

### 2.1. Cow’s Milk

Regarding cow’s milk, its elimination from the diet, in the absence of a balanced diet or vitamin supplementation, is associated with a deficiency of calcium and vitamin D, leading to a reduced bone mineral density and the onset of rickets [20,21]. Other potential nutritional deficiencies involving zinc and B vitamins may also be observed in children [22]. Since cow’s milk also provides essential nutrients like vitamin B12, vitamin A, pantothenic acid (vitamin B5), riboflavin, iodine, and phosphorus, finding a nutritionally dense substitute in the pediatric diet is crucial. Cow’s milk has been found to promote linear growth through the actions of casein and whey on insulin-like growth factor-1 and insulin [23]. Specifically, achieving tolerance to cow’s milk and reintroducing it after outgrowing the allergy resulted in a significant increase in the height-for-age z-score. Therefore, eliminating cow’s milk, in particular, might impact growth. Feeding difficulties in children 2–5 years fed a cows’ milk elimination diet due to food allergy were compared with a control group on an unrestricted diet. Children on an elimination diet showed more picky eating and feeding problems, with picky eating being related to lower weight-for-age z-scores, food refusal/inappetence, constipation and anticipatory gagging associated with feeding difficulties in both groups [24]. Infants with cow’s milk allergy who are formula-fed should avoid intact cow’s milk protein formulas. Extensively hydrolyzed cow’s milk protein and amino acid-based formulas are considered hypoallergenic [25,26]. In most cases of cow’s milk allergy in infants, extensively hydrolyzed cow’s milk protein formulas effectively resolve symptoms. However, a subgroup of children might need amino acid-based formulas [26]. Another option available in Europe is extensively hydrolyzed rice protein-based formula [27]. The tolerance and safety of a new extensively hydrolyzed rice protein-based formula have been demonstrated in managing cow’s milk allergy infants [27,28]. The Diagnosis and Rationale for Action against Cow’s Milk Allergy (DRACMA) summary report recommends against using soy formula for infants with cow’s milk allergy during the first six months of life [13].

### 2.2. Hen’s Egg

Similarly, with egg allergy, an elimination diet may be associated with deficiencies in B vitamins, vitamin D, and selenium [16]. Eggs are rich in high-quality proteins containing all the essential amino acids crucial for tissue growth and repair in children. However, when eggs are removed from a child’s diet due to allergy, there is a risk of inadequate protein intake, hindering growth and development. Also, eggs provide essential nutrients like choline, vital for brain development and neurotransmitter synthesis. Without eggs, children may miss out on choline, with speculation that it could affect cognitive development. Eggs contribute to heart health by increasing HDL cholesterol levels and providing omega-3 fatty acids for heart and brain function. Children without eggs may struggle to obtain these nutrients, impacting their overall well-being. Furthermore, eggs are a source of vitamin D for bone health and immunity and antioxidants for cell protection. Children with egg allergies may lack these nutrients, increasing their susceptibility to oxidative stress. Finally, eggs aid in weight management due to their high satiety levels. Finding alternatives with similar satiety may be challenging for children with egg allergies, affecting their dietary intake and weight management [29]. Careful planning ensures that children with egg allergies receive adequate nutrition for optimal health and growth.

### 2.3. Wheat

Wheat stands out as arguably the most significant food source globally. Across various food cultures, wheat is central to daily meals, featuring prominently in staples like bread, pasta, breakfast cereal, semolina, bulgur, and couscous [30]. Typically, four servings of wheat-based products, such as whole-grain and enriched cereals or bread (e.g., a half cup of hot cereal, a slice of bread, 1 ounce of uncooked pasta), provide over 50% of the recommended dietary allowance (RDA) of carbohydrate, iron, thiamine, riboflavin, and niacin for individuals aged one year and older. Additionally, they are a significant source of folic acid, vitamin B6, and magnesium. The prevalence of wheat allergy is less than 1% [31], and wheat exhibits high cross-reactivity with other cereals, particularly rye and barley. Although oats belong to the same grass family, they are less closely related to wheat. Despite frequent sensitization, children with confirmed wheat allergy typically tolerate ingested oats [32]. Several alternative grains and flours are accessible to individuals with wheat allergies, including rice, corn, oats, barley, buckwheat, rye, amaranth, millet, quinoa, tree nuts, and legumes, if well-tolerated. Notably, some of these wheat flour substitutes derive from other frequently allergenic foods, like nuts, thus necessitating caution. Consequently, these alternative products should be tailored to each individual and contingent upon their tolerance levels as assessed by their allergy specialist [32]. On the other hand, the distinction between wheat allergy and celiac disease is often confounding. While wheat allergy is an allergic reaction to wheat, celiac disease is an autoimmune disorder that occurs in reaction to the ingestion of gluten found in wheat [33]. Whole-grain wheat products also supply fiber to the diet. A gluten-free product will not contain wheat. However, it is important to note that the opposite might not hold true because rye and barley contain gluten. Consequently, a product utilizing barley may lack wheat but still contain gluten. One challenge is that several flours and products are not adequately fortified. Moreover, they might have a lower fiber content. When wheat is eliminated from the diet, alternative sources of these macro- and micronutrients should be provided [32].

### 2.4. Soy

Soy is a nutrient-rich food rich in protein, thiamine, riboflavin, pyridoxine, folic acid, calcium, phosphorus, magnesium, iron, and zinc. Typically, soy is not consumed in large amounts in the diet, so eliminating soy can easily replace the lost nutrients. Exceptions to the minimal impact of eliminating soy on nutritional status include situations where there is a concurrent allergy to cow’s milk or other dietary pattern, such as vegetarianism, which restricts food choices [32]. Soy protein is present in a wide range of products, including baked goods, cereals, crackers, canned tuna and soups, reduced-fat peanut butter, pre-basted meat products, cold cuts, hot dogs, and numerous vegetarian-based items. Removing soy from the diet reduces the variety of commercially available products that offer diverse nutritional and social advantages. The prevalence of soy allergy is 0.4% [34]. Research indicates that most individuals with soy allergies can typically tolerate highly refined soy oil and lecithin [35,36].

### 2.5. Other Foods

Other foods like nuts, fish, shellfish, soy, and wheat can also trigger food allergies in pediatric patients. Despite offering essential nutrients, these foods typically do not make up a significant portion of the daily dietary intake. Many alternative foods provide similar nutrients, allowing for substitutions when dealing with a single allergy. However, meeting nutritional needs can become more complex with multiple food allergies or dietary restrictions, such as vegetarianism. Eliminating these allergenic foods from the diet can potentially lead to further deficiencies in essential micronutrients, macronutrients, and vitamins [16,37,38,39,40,41] (Table 1).

For example, children who are allergic to hazelnuts miss out on vital nutrients like vitamin E, B vitamins, phosphorus, potassium, zinc, and phytosterols found in hazelnuts [42]. Additionally, they miss out on the potential positive effects on cardiovascular health and other health markers associated with hazelnut consumption [43]. Consequently, caregivers must ensure that children with hazelnut allergies receive alternative sources of these nutrients to support their overall health and well-being. Avoiding fish diets has negative psychological effects and excludes an important source of vitamins and omega-3 long-chain fatty acids [44]. The most commonly described nutritional deficiencies in pediatric patients with food allergies include vitamin D, zinc, iron, B vitamins, and fatty acids [40]. The management pathway for each child with food allergies should include more frequent nutritional assessments and growth monitoring than for children without food allergies [41]. This necessitates close collaboration between a pediatric nutritionist and a primary care pediatrician. Periodic monitoring of growth charts and estimating the child’s nutritional needs based on age and compliance with the elimination and substitute diet are essential to mitigate the potential nutritional deficiencies [41]. Dedicated questionnaires such as the Food Frequency Questionnaire (FFQ) can also assess these aspects [45]. The growth chart and nutritional status are strongly interconnected. It has indeed been demonstrated that a slowdown in the growth chart represents the most sensitive indicator of a nutritional deficiency [46]. The timing of child-monitoring visits varies depending on the patient’s age. Without risk factors, it seems reasonable to schedule growth monitoring visits at 1, 2, 4, 6, 9, and 12 months in the first year of life and subsequently every 6–12 months. Additionally, if no pathological features are present, a nutritional assessment should be conducted every 12 months [37] (Table 2).

Within the scope of this evaluation, some blood tests can provide a more specific assessment of this aspect: complete blood count, electrolytes, blood urea nitrogen, lipid profile (total cholesterol, HDL, LDL, triglycerides), creatinine, protein profile (protein electrophoresis, albumin, prealbumin, RBP), iron status (serum iron, ferritin, transferrin), vitamins, and micronutrients [37]. In the context of these serial checks, it is crucial to identify specific risk factors, in relation to which the scheduling of growth-monitoring and nutritional status assessments should be intensified [37]. These risk factors include delayed diagnosis of FA, early onset of such an allergy, presence of multiple food allergies with elimination of most foods from the diet, elimination of high-nutrient-value foods (cow’s milk, eggs) from the diet, poor compliance with diet management (reluctance to expand the diet), extreme food self-restriction, association with atopic diseases (asthma, atopic dermatitis) or chronic diseases [37]. Nonetheless, a substitute diet allows for the introduction of adequate amounts of all nutrients [42]. This diet must not be random but planned by a pediatric nutritionist. Moreover, it has been demonstrated that dietary counselling is associated with improved anthropometric and laboratory nutritional parameters in pediatric patients with food allergies [47]. Pharmacological supplementation can be considered when dietary modifications are inadequate to meet the needs for vitamins, minerals, and trace elements [41].

### 2.6. The Role of the Dietitian

In the context of FA, the dietitian’s role is important in conducting a dietary assessment to ensure adequate energy intake and essential nutrients and provide patient-oriented counselling [48]. It is known that treatment of FA often involves avoiding foods that contain nutrients essential for growth and development [39]. At the same time, not all FAs are alike. Therefore, the presence of a dietitian is always important for FA [48]. At the same time, it is crucial when formulating a diet for breastfeeding and weaned patients. In this case, it is important to determine the nutritional status and food intake of the infant and nursing mother, administering nutritional supplements if necessary. The dietitian plays a role in monitoring and preventing possible related eating disorders in pediatric patients [49]. Additionally, this figure must instruct children and their parents to read hidden allergens on packaged food labels [50]. In particular, the dietitian can play an important role in preventing or correcting nutritional deficiencies in children with FA through training in different skills. Specifically, the verification of the correct consumption of nutrients to achieve recommended levels to maintain a healthy status, monitoring and evaluation of bioavailability and effectiveness of nutrients in foods and supplements, assessment of the nutritional properties of foods and their changes caused by technical and biotechnological processes, assessment of nutritional status for mental and physical health and dissemination of nutrition education [51]. The pediatric allergist and nutritionist will agree on an appropriate approach based on the issues during the nutritional assessment. The assessment of anthropometric indices (weight, length/height, and body mass index) is the most important step in the nutritional assessment because growth is a sensitive indicator of adequate protein and caloric intake. In the presence of nutritional deficiencies, body weight (a sensitive indicator of energy intake) is affected earlier than height. In addition, the patient’s laboratory parameters (protein, lipids, calcium–phosphorus balance, vitamin D, parathyroid hormone, iron and zinc) and nutritional “status” need to be assessed through a targeted, detailed nutritional history to provide comprehensive nutritional guidance [49]. A qualified nutritionist is a core component of the multidisciplinary FA team because, on the one hand, pediatric allergists do not have the opportunity to also devote themselves to these aspects of FA management and, on the other hand, do not have sufficient expertise to carry out theoretical calculation requirements and assessments of each child’s diet [49]. This aim is generally achieved through a traditional method, where patients are predominantly referred by the general practitioner. Collinson et al. developed a specific model, providing more rapid access to the dietitian, reducing the need for general practitioner (GP) and secondary care appointments, and reducing the time taken to receive dietetic input, thereby resolving symptoms more quickly [52]. Success in managing the diet of patients with FA depends on a team combining pediatric allergist and dietitian expertise to educate patients and families on appropriate allergen avoidance and replacement to ensure nutritional needs [49].

## 3. Psychosocial Aspects

Consider a child with a food allergy at the dinner table or party. Often, the child cannot eat the foods that other people consume and perceives parental anxiety around mealtime. The child may be afraid that what he/she takes in may harm his/her health. It is, therefore, not surprising that 25–45% of children with food allergies have feeding difficulties [47]. Feeding difficulties trace their causes to as early as the child’s first months [53]. Specifically, feeding is a learned behavior that develops through an interconnection of motor skills, sensory skills, psychosocial components, and communication with the parent [53]. This physiological learning mode can be interrupted by the onset of an illness, such as a food allergy. It is associated with environmental stress, too [53]. The interruption of physiological learning can interfere not only with the proper learning of the motor sequences that lead the child to feed but also with the child’s motivation and acceptance of the act of feeding [53]. This can lead to frustration, anxiety, and alteration of the parent’s feeding teaching techniques [53]. This negatively affects the child’s participation in family meals [53]. The onset of feeding difficulty generates consequences for both the child and the parents [54]. Specifically, anxiety can play a role in food refusal [54]. The anxious symptomatology can be expressed as a rejection of food based on fear related to previous negative experiences. It involves foods not identified as allergens that the child needs to avoid but cause anxious symptomatology because they are a novelty presented at the table [55]. The anxious symptomatology is not peculiar to only the child [55]. The parent may also be affected, communicating it to the child verbally and non-verbally [55]. Parental anxiety triggers a vicious cycle that reinforces inflexibility and refusal of food in some children, resulting in food selectivity [47]. As part of follow-up visits, the pediatrician must recognize specific red flags, which may lead to the suspicion of feeding difficulties. They are represented by difficulty swallowing, preference for liquids or age-inappropriate foods, vomiting gasps or meal-related vomiting episodes, excessive chewing time and meal consumption in general, acceptance of only a small number of foods in the diet or a small amount of them, irregular timing of main meals with a tendency for numerous snacks throughout the day, and anxious symptoms at mealtime. Having identified a risk factor for feeding difficulties, the pediatrician should request a neuropsychiatric evaluation to confirm the child’s feeding difficulty before it becomes structured. The possible treatment should include the involvement of a multidisciplinary team involving a speech therapist and psychologist [39]. Treatment success largely depends on the parent’s commitment and education about treatment strategies and their application in the home environment and outside [54]. The management of a child with food allergy outside the home environment also appears crucial from a social point of view [53]. The child with a food allergy may have reduced opportunities to participate in peer environments such as playgroups or kindergarten because of the parent’s concern [53]. Regarding accidental exposure to the food allergens to which the child is allergic [53], this practice also limits the child’s ability to “learn to eat” with associated developmental skills [53]. In addition, some patients do not outgrow food allergy during early childhood. In these individuals, there is a significant association between food allergy and pathological connotations at the emotional and social levels [56], including anxiety and depression [8,57]. This observation is related to a further association between restrictive eating patterns and the onset of anorexia and bulimia nervosa in adolescents and young adults [58,59]. Recent evidence has also demonstrated this correlation in the context of food allergy [60] and the possibility of the association between food allergy, bodily dispersion, and eating disorders [61]. Food allergy has a social connotation as well [62]. A total of 53% of families with children with food allergy have abandoned the restaurant table on at least one occasion, 89% avoid specific restaurants, 57% have changed their vacation planning on at least one occasion, 69% decide to accompany their child on school-organized trips, and 25% have changed their child’s school [62]. Food allergies thus impact the quality of life of affected individuals and their caregivers. Many factors can affect a child’s quality of life, such as age, severity of the food allergy, and allergy history [63]. Rubeiz et al. focused on the concept of resiliency in these children. It means maintaining a positive developmental trajectory in the face of adversity. Healthcare providers bolstering resiliency factors during routine clinical care can optimize the quality of life of children with food allergy and their caregivers. Resilience in patients and parents should be obtained in many ways. First, education on the risk of allergen exposure is important. Second, self-efficacy should be enhanced in communicating anaphylactic symptoms, using autoinjectors, and assertiveness regarding allergen avoidance. Third, determining solutions is needed to assess social concerns and barriers to full engagement in developmentally important activities and problem-solving with families. Last, reflection with patients and families about the strengths that could help them navigate the challenges that food allergy causes every day [64].

## 4. Personalized Approaches in Managing Food Allergies: Enhancing Patient Well-Being

Noncompliance with dietary and medical recommendations for food allergies is prevalent despite the potentially life-threatening consequences [65]. Among adolescents and young individuals, full adherence to exclusion diets and carrying adrenaline autoinjectors may be as minimal as 17–21% [66]. Factors influencing adherence encompass age, race, social networks, financial status, challenges with food labeling comprehension, bullying, mental health assistance, and smoking habits [65]. Globally, access to allergy services varies significantly, often resulting in prolonged wait times to consult a healthcare provider or even a complete lack of support [65]. Specifically, there is a shortage of specialized psychologists to aid individuals with food allergies [67]. Upon diagnosis, families and caregivers may require considerable time to grasp the implications of the food allergy, often necessitating repetitive education to grasp crucial information. This educational process can be hindered by false information, inconsistent guidance, and the influence of social media [68]. Strategies proven to enhance adherence include joining patient support communities, possessing a documented anaphylaxis management strategy, and aiding adolescents in comprehending the seriousness of anaphylactic reactions [66]. Where accessible, psychotherapy can lead to enhanced treatment engagement and management [69]. Managing food allergies, particularly egg and milk allergies and peanut and tree nut allergies, highlights the importance of personalized approaches. While complete avoidance of allergenic foods may not always be necessary, partial avoidance coupled with the introduction of baked forms has shown promise in expanding dietary options and improving the quality of life of affected individuals [65,70]. For egg and milk allergies, completely avoiding all forms may not always be necessary [70]. A more personalized approach with only partial avoidance of the culprit food has already been successfully implemented for many children with cow’s milk and hen’s egg allergy [65]. Baked food allergens may be tolerated in up to 70% of milk- and egg-allergic children, expanding their diet and improving their quality of life [71,72,73,74,75]. Extensive heating, such as occurs during baking, reduces protein allergenicity by denaturing the conformational epitopes present within the food. Baked forms of egg and milk include individual muffins, cakes, and rolls baked at 180 °C for 25–30 min within a wheat or carbohydrate complex, ensuring that the center of the individual baked product is completely cooked and not wet or soggy in the middle. Since introducing baked forms does not come without risk, these allergens should be introduced with guidance and support from allergy specialists [76]. Tolerance of baked eggs has been linked with early resolution of egg allergy, but it remains unclear whether early introduction and regular consumption of baked milk and/or eggs accelerates allergy resolution [77]. Peanut and tree nut allergies tend to not be outgrown, with 9 and 20% of those allergic to any nut developing tolerance over time [71]. Considering the enduring nature of primary nut allergy, prioritizing pharmacological interventions to alter its natural course (or at least mitigate the risk of severe reactions) is essential. The most promising outcomes have been achieved through oral and sublingual immunotherapy [78]. By combining personalized dietary management with targeted pharmacological strategies, healthcare professionals can strive to enhance the well-being of patients living with FA.

## 5. Conclusions

FA represents a significant public health concern affecting both children and adults. Despite advancements in specific immunotherapy, allergen avoidance remains pivotal in managing FA. Accidental ingestions are common, and although fatal reactions are rare, they impose substantial psychological burdens on individuals and their families. The nutritional implications of FA are extensive. Due to allergen elimination diets, patients often face energy intake challenges, leading to growth delays and nutrient deficiencies, especially with cow’s milk, egg, and wheat allergies, which provide essential nutrients. Moreover, psychosocial factors significantly impact FA management, with children experiencing feeding difficulties, anxiety, and social limitations affecting their quality of life and that of their caregivers. Personalized management strategies, like partial avoidance and introducing baked forms, promise to expand dietary options and improve quality of life. Pharmacological interventions such as oral and sublingual immunotherapy potentially alter FA’s natural course. However, noncompliance with dietary and medical recommendations remains a significant challenge due to various factors, like age, race, social support, financial status, and mental health assistance. Inconsistent access to allergy services globally contributes to diagnosis delays and inadequate support, compounded by a shortage of specialized psychologists. Personalized approaches, like partial avoidance and introducing baked forms, offer promising avenues but must be cautiously implemented under allergy specialists’ guidance. Ongoing research into pharmacological interventions underscores the need to prioritize interventions to alter FA’s course and mitigate severe reactions. Integrating personalized dietary management, pharmacological strategies, and comprehensive allergy services can enhance outcomes and well-being in individuals with FA. Patient education, support communities, and anaphylaxis management strategies are vital to effective FA management. Collaboration among healthcare professionals, caregivers, and patients is essential for achieving optimal outcomes and improving FA management globally.

## Figures and Tables

**Table 1:** The main foods that are implicated in food allergies and the potential nutritional deficiencies (modified from [37]).

**Milk**	Vitamin A, vitamin D, riboflavin, acid pantothenic, vitamin B12, calcium, phosphorus, iron, zinc
**Egg**	Riboflavin, pantothenic acid, vitamin B12, biotin, selenium
**Soy**	Thiamine, riboflavin, pyridoxine, acid folic, calcium, phosphorus, magnesium, iron, zinc, group B vitamins
**Wheat**	Thiamine, riboflavin, niacin, iron, acid folic, vitamin B12
**Peanut**	Vitamin E, niacin, magnesium, manganese, chromium, calcium, iron, vitamins of group B
**Fish and shellfish**	Zinc, iron, omega-3

**Table 2:** A flow chart of the nutritional and psychological health of a child with food allergy *.

**Time 0 (food allergy diagnosis)**	Weight growth assessment by the physician Pediatric nutrition visit Child neuropsychiatric visit
**If age < 1 year: 1, 2, 4, 6, 9, 12 months of life** **If age > 1 year: every 6–12 months of life**	Weight growth assessment by the physician
**Every 6–12 months, starting from time 0**	Pediatric nutrition visit, in association with blood tests (blood count, azotemia, electrolytes, creatinine, lipid profile (total cholesterol, HDL, LDL, triglycerides), profile protein (protein electrophoresis, albumin, prealbumin, RBP), martial structure (serum iron, ferritin, transferrin), vitamins and micronutrients)
**Every 12 months, starting from time 0**	Child neuropsychiatrist visit

* Any variations in timing should be attributed to the presence of the following risk factors: delayed diagnosis of FA, onset of such an allergy at an early age, presence of multiple food allergies requiring the elimination of most foods from the diet, elimination of nutritionally valuable foods from the diet, poor compliance with dietary management, extreme food self-restriction, association with atopic diseases (asthma, atopic dermatitis), or with chronic diseases.

## Data Availability

Not applicable.
